# Bridging the Gap Between Science and Practice: Research Collaboration and the Perception of Research Findings

**DOI:** 10.3389/fpsyg.2021.790451

**Published:** 2021-12-16

**Authors:** Hadjar Mohajerzad, Andreas Martin, Johannes Christ, Sarah Widany

**Affiliations:** ^1^German Institute for Adult Education (LG), Bonn, Germany; ^2^Department of Educational Sciences, University Potsdam, Potsdam, Germany

**Keywords:** science and practice relationship, research collaboration, research findings, survey experiment, Bayes factor method

## Abstract

Research collaboration promises a useful approach to bridging the gap between research and practice and thus promoting evidence-informed education. This study examines whether information on research collaboration can influence the reception of research knowledge. We assume that the composition of experts from the field and scientists in a research team sends out signals that influence trust in as well as the relevance and applicability of the finding. In a survey experiment with practitioners from the field of adult education the influence of different research team compositions around an identical finding is tested. The results show overall high trust, relevance and applicability ratings with regard to the finding, regardless of the composition of the research team. We discuss the potential importance of additional information about research collaborations for effective knowledge translation and point out the need for more empirical research.

## Introduction

The question of whether and how scientific evidence can make educational systems and professional action in educational systems more efficient has been a focus of educational science discourse and research programs for the last decades (Hargreaves, 1999; Slavin, 2004; Nelson and Campbell, 2017; Pellegrini and Vivanet, 2021). In the United States, for example, the No Child Left Behind Act (2002)^1^ required programs and teaching methods to be based on scientifically derived research results. The adult education legislation of the United States, Workforce Innovation and Opportunity Act (2014), includes, among other things, the claim to evidence-based education. Practitioners in adult education should thus be supported with scientific knowledge in order to professionalize their skills. Professionalism requires individuals to act with the best available (research) knowledge and to reflect on their actions to improve their professional practice (Thomm et al., 2021). In Great Britain, Hargreaves (1998) in particular called for a stronger evidence-based education at a Teacher Training Agency meeting. Evidence-based approaches in education policy are linked to the expectation that the findings of empirical educational research can serve as a knowledge basis for rational decisions and thereby improve the performance of education systems (e.g., Peurach and Glazer, 2012; Tseng et al., 2017). Repeatedly, programs of evidence-based educational reform challenge empirical educational research (Biesta, 2007; Schrader et al., 2020) to communicate research findings to policymakers and practitioners, thereby contributing to improving actions and decisions (Penuel et al., 2020). However, there is a broad consensus that the communication of research findings and their application in the broad field of education has not been satisfactorily implemented to date (Broekkamp and Hout-Wolters, 2007; Kinyaduka, 2017; Tseng et al., 2017). This much-cited and multifaceted gap between research and practice has led to a controversial debate about evidence-based approaches in education. For example, the communication of research findings is criticized that it resembles a one-way approach that neglects the need to address practice issues (Tseng et al., 2017). Hargreaves (1999, p. 246) also refers to the contextuality of policy and practice decision-making processes in which scientific evidence is one factor in a complex structure. He therefore suggests using the term evidence-informed instead of evidence-based policy or practice to account for the quality of evidence and other (constraining) contextual factors in these fields of action.

The debate has been going on since the origins of educational science in the eighteenth century, and the relationship of educational science and educational practice (and the gap in between) has become a constitutive element, if not an academic feature, of the field (Biesta, 2007). Roughly summarized, the current discussion on the causes of this gap and solutions to it develops along two strands that show some cross-connections (Broekkamp and Hout-Wolters, 2007). The first strand pursues the argumentation that the results of educational research do not meet the needs of policy and practice with regard to the relevance and quality of content and applicability. The second strand focuses on the requirements and conditions for the reception and use of scientific research in practice.

With regard to the first strand, one needs to consider the quality and quantity of evidence in a given field of research. In the field of adult education—which will be considered in this paper in particular—empirical research activities have only been intensified in the last 50 years (Born, 2011; Rubenson and Elfert, 2015) and there is an ongoing debate about whether methodological approaches are fit to deliver the demand for evidence in the competitive field of empirical educational research (Boeren, 2018; Daley et al., 2018). A large number of non-empirical research and an altogether expandable state of research^2^ can therefore be a valid factor contributing to the gap between adult education research and practice. The gap between science and practice can be described with various formulas: farewell to the ivory tower of science (Hayes and Wilson, 2003), reference to basic research with little practical relevance (Siebert, 1979; Hargreaves, 1998) and a distinction of the two cultures (Fox, 2000; Ginsburg and Gorostiaga, 2001).

Regarding the second strand, research knowledge does not seamlessly find its way into practice or policy (Tseng et al., 2017). Target groups must be able to make sense of information by reading, interpreting, and applying research knowledge to their situation in order to make necessary decisions (Brown et al., 2017). Rather, there is a consensus in empirical educational research that evidence-informed education requires the integration of knowledge gained from experience and professionalization with systematically acquired research knowledge, whereby the different types of knowledge are not replaced, but have a reciprocal influence (Ratcliffe et al., 2005) and may depend on specific professional culture and practice (Booher et al., 2020). Concepts and designs to transfer research knowledge into practice, account for reciprocity considering specific contexts of use and professional development as well as dedicated resources (Cordingley, 2009; Wollscheid et al., 2019). The literature offers a range of solutions to build bridges between science or theory and practice in adult education (Siebert, 2011). Solutions are, for example, activities and the provision of resources for “linking theory and practice” in adult education to extend the scope to the audience of educational practice as well (Merriam and Bierema, 2014). Other solutions to reduce the science-practice-gap include skills development in universities (Schön, 1995; Hargreaves, 1998; Fox, 2000; Jütte and Walber, 2015) and scientific education (Kennedy, 1997; Jütte and Walber, 2015). Furthermore, forms of communication and institutionalization are addressed, e.g., knowledge translation by the Texas Adult Literacy Clearinghouse (St. Clair, 2004), the use of meaning making language (Roessger, 2017), trialogues between science, practice and politics (Robak and Käpplinger, 2015) or “Jour fixe” as discussion and lecture events (Dausien et al., 2016). Other approaches to bridge the science-practice-gap combine both strands by incorporating relations between science and practice in the research process: (research) workshops, e.g., problem definition workshops, consulting workshops and interpretation workshops, mentoring exchange relationships and practitioner-based research (Dirkx, 2006; Jütte and Walber, 2015) or research collaboration (Penuel et al., 2020; for the adult education sector, cf. Siebert, 1979).

Our study focuses on the role of such collaborations to bridge the gap between science and practice. Research collaboration or research practice partnerships are discussed as a promising approach to enhance the use of evidence in practical decision making in education and there is a demand for studies on their conditions of success and outcomes (Coburn and Penuel, 2016; Wentworth et al., 2017). We approach the topic of research collaboration as a potential solution for bridging the science-practice-gap from a large-scale dissemination perspective. We are interested if research collaboration between adult education practice and science has an effect on practitioners’ perception of research findings. In this sense, we are not concerned with the use and application of knowledge within specific research collaborations but investigate on the more general level of dissemination of research output and its contribution to knowledge transformation (Cordingley, 2009). More specifically, we are interested if information on one quality aspect of research collaboration—the composition of scientists and practitioners in the research team—affects the reception of research findings. The central questions are: How does collaboration between science and practice within research processes in the field of adult education influence practitioner’s (1) trust in research findings, (2) attributed relevance of research findings and (3) applicability of these findings? The analysis is guided by system theory and signal theory. Derived hypotheses are tested with data from a survey experiment, testing varying research-practitioner-constellations, applying Bayes factor methods. The paper has the following structure: We first derive the hypotheses based on theoretical considerations and research results related to the concept of practice research collaboration (section “Bridging the Gap Between Science and Practice Through Collaboration—Theoretical Perspectives”). Then we present the methodology, design, data basis and method of analysis (section “A Survey Experiment on The Perception of Research Knowledge in a Collaborative Setting”). The presentation of the results (section “Results”) is followed by a discussion (section “Discussion”).

## Bridging the Gap Between Science and Practice Through Collaboration—Theoretical Perspectives

In the scientific literature the relationship between (adult education) science and practice is often conceptualized theoretically using hermeneutic procedures to explain the *tension* between science and practice. There is little data on what the relationship actually is (St. Clair, 2004). The tension is a structurally determined distance between the two systems (e.g., Feuer, 2006). Thus, there are numerous reasons for this distance, and just as many challenges arising from it (e.g., Fox, 2000; Broekkamp and Hout-Wolters, 2007). From the perspective of practitioners, research questions are of little *practical relevance*. They consider their role as “experimental objects” and see themselves as barely involved in research or unable to apply research results (e.g., Siebert, 1979; Fox, 2000). Practice often perceives science as an alien theory that is generated in an ivory tower—one that seeks answers to questions of no relevance to practice and that leaves adult education staff and institutions alone with their daily questions (Faulstich, 2015). Practitioners complain that research knowledge does not meet the needs of practitioners and that research does not offer practice-relevant knowledge on specific topics (Dean, 1999). The reception of research knowledge requires a clear presentation and linguistic comprehensibility on the part of the scientific community (St. Clair, 2004; Christ et al., 2019) and an awareness of the practical relevance of scientific questions (St. Clair, 2004).

In general, there is very little research on the extent and quality of practitioners’ use of research knowledge in their practice. The sparse findings are rather sobering. K-12 teachers, for example, show a low engagement with scientific evidence in order to inform their teaching practices (Booher et al., 2020). Surveys with adult education providers show that they perceive the intensive exchange with science as useful. At the same time, they feel that scientific research is not sufficiently interested in practice-relevant issues (Christ et al., 2019). However, if they consider research knowledge as relevant, they are more likely to apply this knowledge (Weiss and Weiss, 1981, as quoted in Huberman, 1994) and even change their practice accordingly (St. Clair, 2004). Thus, with major reservations against its relevance and applicability, scientific knowledge is nevertheless recognized as suitable problem solution by practice (St. Clair, 2004; Christ et al., 2019). Practitioners even demand resources from research to deal with everyday problems (Fox, 2000).

The aim of informing and improving practice has an impact on the type of research and the methodology applied (St. Clair, 2004). In order to reduce the structural distance between science and practice, empirical research demands collaboration in the sense of open and collaborative interaction in the research process (see *design-based research approach* e.g., Anderson and Shattuck, 2012 or see *use-inspired basic research* based on Stokes, 1997; e.g., Feuer, 2006; Goeze and Schrader, 2011). In adult education research, too, one of the central issues of the relationship of research to practice is the low level of exchange with practice on the research topics (e.g., Siebert, 1979; Faulstich, 2015). Huberman (1994), for example, believes that it is necessary for researchers and practitioners to work together on knowledge production. Therefore, various forms of research collaboration can contribute to reduce the gap. We understand *research collaboration* as the collaboration between scientist and practitioner. Whether research knowledge is actually used, however, is finally decided on the practical side. Practitioners actively determine “what is useful and how it is useful” (St. Clair, 2004, p. 238), and ultimately whether they will deal with research-based information or not. After all, the main barriers are the perception of *applicability* and *relevance* of research knowledge, as well as lack of *trust* in science (van Schaik et al., 2018).

### Trust in Science by and in Practice

In the public and in science itself, collaboration between research and practice is sometimes problematized with regard to the openness and independence of research (Besley et al., 2017). If, however, practice is supposed to rely on research knowledge in the sense of evidence-informed education, then practitioners must be able to trust the communication of scientists (Kennedy, 1997). Indeed, trust is an essential element that supports evidence-based decision making within a researcher-practitioner relationship (Wentworth et al., 2017). Trust in science and scientists as a construct has both rational and emotional components. General assessments, for example, show different levels of trust depending on the level of education and political or religious attitudes (Nadelson et al., 2014). Trust attitudes can also vary depending on context and situation, leading to different expectations and different levels of trust in science, on the interests and logics of action in the practical domain (Resnik, 2011) on the practice context and culture (e.g., subjective beliefs and values) or science context (e.g., controversial research findings, researchers’ interests, etc.). However complex and multifaceted, trust is a necessary condition for the transfer of scientific knowledge (Mohajerzad and Specht, 2021). Bormann (2012) argues that the acquisition of scientific knowledge through educational reporting leads to an increase of complexity, which can in turn be reduced by trust.

Trust reduces complexity by absorbing uncertainty. In other words, when it is uncertain how an event will occur (contingency), trust refers to another’s ascertainable future action, thereby opening up possibilities for action that would be unlikely without trust (Luhmann, 1988, 2014). In this sense, trust does not result from information about the trustworthiness of an actor but replaces this missing information and thus enables action. Following Luhmann, we assume that trust is a function that arises from contingency and that allows us to act despite uncertainty (2014). Scientists select research questions, theoretical approaches, hypotheses, research designs and methods from a wide variety of possibilities without any guarantee that the research activity will lead to an applicable, robust result. The research process is hardly comprehensible to outsiders and leads to a fundamental uncertainty. Therefore, the application of research results requires trust. However, as uncertainty applies to all social interactions, information about practitioners’ participation in the research team will not reduce uncertainty. We therefore assume that research practice collaboration has no influence on trust in research results, leading us to our first hypothesis:

*Hypothesis 1:* Trust in research knowledge does not depend on research practice collaboration during the research process.

### Signals for Practical Relevance and Applicability of Research Knowledge

Signal theory shows yet another way to conceptualize uncertainty in social interactions. As in Luhmann’s approach, signal theory assumes missing or insufficient information as well as information asymmetry between actors. In such a situation, signals can create certainty for decisions and actions. The starting point of the approach is a situation that is typical for game theory: Actor 1 and actor 2 can gain a benefit if actor 2 does something for or with actor 1. However, the prerequisite for the benefit of actor 2 is that actor 1 has a good (k). Actor 1 benefits in any case when actor 2 acts, regardless of whether he has (k) or not. For actor 2, it is therefore important to know whether actor 1 actually has (k). However, she cannot be sure of this. A signal whether actor 1 actually has the good (k) can solve this fundamental dilemma. In order for this signal to give a reliable indication of (k), the cost of the wrong signal that actor 1 has (k), although she does not have it, must be higher than the benefit that actor 1 receives when actor 2 acts based on wrong information (Gambetta, 2009). The next two sections show how signals can illustrate the effect of collaboration between science and practice on the willingness of practice to apply research results.

#### Practical Relevance of Research Knowledge

If science commits to the idea of evidence-informed practice, it is important for researchers that their results are applied in practice. However, the representatives of practice decide whether scientific knowledge is suitable for practice or not. Practitioners can only guess the importance that practical relevance and applicability has had in the research process. Meanwhile, they only benefit if the findings help them solve practical problems or gain other practically relevant advantages. In this situation, the signal emanating from collaboration between practice and science can break the information asymmetry between science and practice. Contrary to scientists who actually conduct application-relevant research, scientists who conduct pure basic research without any practical relevance will have difficulties finding and keeping collaborative practice partners. Therefore, collaborative research can be a valid signal for practice-relevant research, and sets itself apart from detached research coming out of the ivory tower (e.g., Faulstich, 2015).

While the participation of practitioners assures practical relevance, the participation of scientists ensures that scientific standards are met (Feuer, 2006). This results in two basic assumptions about how the signal of collaboration can affect the assessment of practical relevance. A continuous effect should occur if the validity of the signal for practical relevance increases with the number of practitioners in the research project. In contrast, a discrete effect would be expected if collaboration between scientists and practitioners signals practical relevance in any case, regardless of how many practitioners are involved.

*Hypothesis 2*
*2a*: The higher the proportion of practitioners in the research process, the higher the practical relevance of research knowledge.
*2b:* In the case of research collaboration between scientists and practitioners, the practical relevance of research knowledge is higher than without research collaboration.

#### Applicability of Research Knowledge

The Signal theory is also valid with regard to the applicability of research results. While practical relevance is an important precondition for the transfer to practice, practical relevance does not necessarily mean that the knowledge generated will actually be implemented. Therefore, the knowledge must also be applicable. Research is applicable if it is tailored to practice, i.e., if research deals with problems and experiences of practice. Producing scientific knowledge through collaboration can be a beneficial condition for dissemination (van Schaik et al., 2018). Another position assumes that research is applicable if it is possible to generalize research knowledge and thus transfer a specific solution to a broader or different context (Ercikan and Roth, 2014). However, neither of these goals can be achieved without compromises. While the generalizability of research results by the use of scientific methods and standards is highly desirable from a scientific perspective, findings from applied research that are produced by practitioners themselves, as in action research, may be more applicable (Kuhn and Quigley, 1997). Structures in research signaling an engagement of practitioners by collaboration or by action research, could thus influence practitioners in terms of using research knowledge (Henson, 2001; Levin and Rock, 2003). This leads to a set of three possible theses for the assessment of applicability. In the first thesis, we assume that the most important factor for applicability is the focus on practical needs rather than the emphasis on generalizability and advancement of theories. A higher proportion of practitioners involved in the research process should signal a higher applicability of the produced knowledge. In the second thesis, we assume that practitioners perceive research results as more applicable if both generalizability and practicability are signaled. In this case, collaboration signals applicability, regardless of the proportion of scientists and practitioners involved in the research project. In the third thesis, we assume that generalized knowledge generated by rigorous scientific standards, such as validity of data or experimental designs, is produced by scientists in particular. Since research is particularly applicable when research knowledge is generalizable (Ercikan and Roth, 2014), we assume that scientists involved in the research process signal generalizable research knowledge and thus a high applicability. Thus, the higher the proportion of scientists, the higher the assessment of applicability.

*Hypothesis 3*
*H3a:* The higher the proportion of practitioners in the research process, the higher the perception of the applicability of research knowledge.
*H3b:* In case of a research collaboration between scientists and practitioners, the perception of the applicability of research knowledge is higher than in the case of no research collaboration.
*H3c*: The higher the proportion of scientists in the research process, the higher the perception of the applicability of research knowledge.

## A Survey Experiment on the Perception of Research Knowledge in a Collaborative Setting

### Data and Design

In order to test the impact of research collaboration opportunities in terms of perception and trust in research knowledge, we use data from the German wbmonitor 2019. Wbmonitor is an annual online survey of adult education and training providing organizations in Germany conducted in cooperation with the Federal Institute for Vocational Education and Training (BIBB) and the German Institute for Adult Education---Leibniz Centre for Lifelong Learning e.V. (DIE). The survey annually collects information on the economic situation, staff and services of the organizations as also information on annually changing thematic focal points.^3^ In 2019, 18,050 organizations were invited to participate in the survey between May and June. Our analysis is based on the sample of 1,551 organizations with valid survey participation (Christ et al., 2020), i.e., 1,551 respondents within these organizations. The sample covers different types of organizations : private commercial providers (22%), private non-commercial providers (15%), institutions affiliated with churches, parties, trade unions, non-profit associations or foundations (19%), adult education centers (16%), business-related institutions (10%), vocational schools (8%), educational institutions of companies (3%), universities and universities of applied sciences (3%) and other types of public institutions (2%). The questionnaire is usually answered by persons with leadership and planning roles within the organizations surveyed.^4^

**FIGURE A1:**
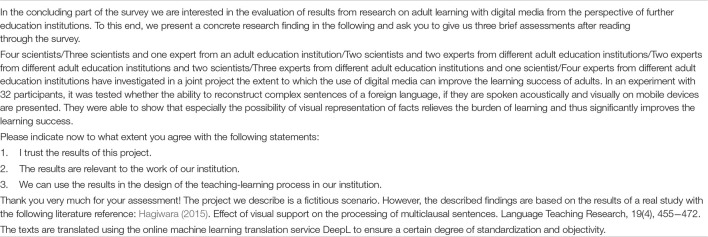
Treatment and Outcomes.

For our analysis we use data of a survey experiment that was conducted in addition to the regular survey program in 2019. Survey experiments combine the advantages of randomized experiments with the possibilities of large representative surveys. This design enables causal relationships to be identified and at the same time guarantees a high internal and external validity (Auspurg and Hinz, 2015). Within the experiment, which was placed at the end of the regular survey program, each of the respondents was presented a vignette containing a research result produced by a specific research team (see Figure A1 for the research design). While the presented research result was identical for all of the respondents, the composition of practitioners and scientists in the research team was varied between six groups. The distinction was made between four scientists, three scientists and one expert (working in an adult education providing organization), two scientists and two experts and vice versa, three experts and one scientist and lastly four experts. The vignettes were allocated to the wbmonitor population by a random split into six groups, before the organizations were invited to participate in the survey. The sizes of the six splits in the analyzed sample differ slightly. They are between 15 and 17% of the total sample (see Table 1).

**TABLE 1:** Sample split.

Group	Sample size	%
Four scientists	258	16.63
Three scientists and one expert	272	17.54
Two scientists and two experts	257	16.57
Two experts and two scientists	260	16.76
Three experts and one scientist	245	15.80
Four experts	259	16.70
Total	1,551	100.00

*Own calculations using wbmonitor 2019.*

Three items ask the respondents (1) whether they trust the presented research result, (2) whether the results are relevant to the field of activities in their organization and 3) whether the results can be applied in their organization (see Figure A1 for questions in the survey). The three items were surveyed using a five-point Likert scale, marked with 1 = “++,” 2 = “+,” 3 = “0,” 4 = “-” and 5 = “–” and verbalized endpoints (++ “agree completely,” – “do not agree at all”).

### Analytical Strategy

We model the effect of different compositions within research practice collaboration on the perception of research results using Bayesian variance analyses. While frequentist statistics with the *p*-value can only indicate the dependent probability P(D| H0) that the data D occur under validity of the null hypothesis, the probability P(H| D) is of interest, i.e., how probable is a hypothesis among the data obtained. This can be calculated with Bayesian statistics, because with the Bayesian theorem both probabilities can be related to each other (Hoijtink et al., 2019):


$$B\,F_{10}=\frac{P\,\left(D|H_{1}\right)}{\text{P}\,\left(\text{D}|H_{0}\right)}$$

The so-called Bayes Factor (BF) expresses quantitatively to what extent the data obtained speak in favor of a zero model/hypothesis or an alternative model/hypothesis. Although frequentist statistics will be used to calculate a *p*-value for whether the null hypothesis should be rejected, quantifying the *p*-value as the strength of the data against the null hypothesis is linked with restrictions. Analyses of Lin and Yin (2015) show that even if the null hypothesis is rejected, there is still a probability of about 20% that the null hypothesis is true. Therefore, the Bayesian posterior probability of the null hypothesis is appropriate for examining how strongly the data support the null hypothesis (Lin and Yin, 2015). Our first hypothesis is therefore tested using the Bayesian posterior probability of the null hypothesis. Another advantage of Bayes’ theorem is that the BF can be used to test a set of hypotheses (Hoijtink et al., 2019). BF then selects the best suitable hypotheses. Since we have two sets of hypotheses (2a and 2b resp. 3a, 3b, and 3c), we can use Bayesian variance analysis to model our hypotheses against each other. We perform the data analysis with the Bayes-Factor-Package *bain*^5^ in the statistics program R (Gu et al., 2018). This follows in the tradition set by O’Hagan (1995). We present our results based on the recommendation for reporting results from Hoijtink et al. (2019, p. 553–554).

## Results

Table 2 provides an overview of all dependent variables and their main summary statistics that we calculate using the total analytical sample of all variables. Across all groups, it is evident that research knowledge is trusted (*M*-values: 2.16–2.25) and perceived as practically relevant (*M*-values: 2.27–2.53) and applicable (*M*-values: 2.48–2.57).

**TABLE 2:** Descriptive statistics of dependent variable.

Dependent variables	Group	M	SD	Range	N
Trust	Four scientists	2.25	0.86	1–5	254
	Three scientists and one expert	2.16	0.72	1–5	267
	Two scientists and two experts	2.21	0.79	1–5	247
	Two experts and two scientists	2.19	0.83	1–5	257
	Three experts and one scientist	2.17	0.89	1–5	240
	Four Experts	2.20	0.82	1–5	251
Practical relevance	Four scientists	2.27	0.96	1–5	253
	Three scientists and one expert	2.50	0.92	1–5	267
	Two scientists and two experts	2.42	0.96	1–5	248
	Two experts and two scientists	2.48	1.04	1–5	258
	Three experts and one scientist	2.52	1.03	1–5	239
	Four experts	2.53	0.98	1–5	251
Applicability	Four scientists	2.57	0.98	1–5	253
	Three scientists and one expert	2.56	0.93	1–5	266
	Two scientists and two experts	2.48	0.97	1–5	247
	Two experts and two scientists	2.56	0.99	1–5	258
	Three experts and one scientist	2.57	1.03	1–5	239
	Four experts	2.57	0.97	1–5	251

*Own calculations using wbmonitor 2019.*

To examine our three hypotheses (or sets of hypotheses), Bayesian analyses of variance were performed. The posterior distribution of the Bayesian analysis summarizes the information in the data and the prior distribution in relation to the population mean of each of the groups in the ANOVA. The Bayes factors vs. *H_u_*^6^ and the Bayesian probabilities are displayed in the table below. The Bayesian probabilities—also called posterior probabilities—quantify the support for hypothesis (e.g., *H_0_*) and *H_u_* after the data is observed (Hoijtink et al., 2019). Hence, P(*H_0_*| data) can be regarded as the Bayesian error probability if *H_u_* is chosen as the preferred hypothesis, and P(*H_u_*| data) is the Bayesian error probability if *H_0_* is chosen as the preferred hypothesis. The ratio of these probabilities (the posterior odds) can be calculated using the BF and prior odds over $\frac{P\,\left(H_{0}|d\,a\,t\,a\right)}{P\,\left(H_{u}|d\,a\,t\,a\right)}=B\,F_{0\,u}\times \frac{P\,\left(H_{0}\right)}{P\,\left(H_{u}\right)}$(Hoijtink et al., 2019, p. 544).

### Trust in Science by and in Practice

In our first hypothesis we assumed that trust in research knowledge does not depend on research practice collaboration during the research process. Table 3 shows the results from testing our hypotheses on trust in research knowledge. Two hypotheses corresponding to the Bayesian variance analysis are displayed:^7^

**TABLE 3:** Bayesian informative hypothesis testing (ANOVA) of Trust.

Hypothesis	BF._ɑ_	Posterior probability
*H_1_*	602430.05	1.00
*H_u_*		0.00


$$H_{1}:\mu_{4\,S}=\mu_{3\,S\,1\,E}=\mu_{2\,S\,2\,E}=\mu_{2\,E\,2\,S}=\mu_{3\,E\,1\,S}=\mu_{4\,E}$$

$$H_{u}:\mu_{4\,S},\mu_{3\,S\,1\,E},\mu_{2\,S\,2\,E},\mu_{2\,E\,2\,S},\mu_{3\,E\,1\,S},\mu_{4\,E}$$

As can be seen, *BF*_1*u*_ = 602430.05, that is, the support for*H*_1_ is still 602 430.05 times larger than for *H_u_*. The Bayesian error probability associated with preferring *H_1_* equals zero. *H_1_* is the preferred hypothesis. This means we can assume that trust in research knowledge is not dependent on research practice compositions in the research process.

### Practical Relevance of Research Knowledge

Second, four hypotheses for the variables of practical relevance are evaluated, which are firstly, the higher the proportion of practitioners in the research process, the higher the practical relevance of the research knowledge (2a) and secondly, when there is research collaboration between researchers and practitioners, the practical relevance of the research knowledge is higher than without research collaboration (2b). Following the Bayesian approach, these hypotheses were again tested against the *H_0_*, that there is no difference, and against the *H_u_*, that there is no relationship between the constellations.


$$H_{0}:\mu_{4\,S}=\mu_{3\,S\,1\,E}=\mu_{2\,S\,2\,E}=\mu_{2\,E\,2\,S}=\mu_{3\,E\,1\,S}=\mu_{4\,E}$$

$$H_{2\,a}:\mu_{4\,S}<\mu_{3\,S\,1\,E}<\mu_{2\,S\,2\,E}=\mu_{2\,E\,2\,S}<\mu_{3\,E\,1\,S}<\mu_{4\,E}$$

$$H_{2\,b}:\mu_{4\,S}=\mu_{4\,E}<\mu_{3\,S\,1\,E}=\mu_{2\,S\,2\,E}=\mu_{2\,E\,2\,S}=\mu_{3\,E\,1\,S}$$

$$H_{u}:\mu_{4\,S},\mu_{3\,S\,1\,E},\mu_{2\,S\,2\,E},\mu_{2\,E\,2\,S},\mu_{3\,E\,1\,S},\mu_{4\,E}$$

The Bayes factors vs. *H_u_* and the posterior probability are displayed in Table 4. As can be seen, *H_0_* is supported more than *H*_2*a*_, *H*_2*b*,_ and *H_u_*. The posterior probability *H_0_* has the highest posterior model probability (0.96) and thus is the best suitable hypothesis of the set of hypotheses. We can therefore not accept any of our hypotheses on the influence of research compositions on the perception of practical relevance. This is because the null hypothesis is confirmed, i.e., practical relevance in research knowledge is not dependent on research practice compositions in the research process.

**TABLE 4:** Bayesian informative hypothesis testing (ANOVA) of practical relevance.

Hypothesis	BF._ɑ_	Posterior probability
*H_0_*	327530.63	0.97
*H*_*2a*_	2.78	0.00
*H*_*2b*_	8500.21	0.03
*H_u_*		0.00

### Applicability of Research Knowledge

To examine the third set of hypotheses with regard to the applicability of research knowledge, three hypotheses were contrasted: The higher the proportion of practitioners in the research process, the higher the perception of applicability of research knowledge (H3a), in case of research collaboration between researchers and practitioners, the perception of applicability of research knowledge is higher than in case of no research collaboration (H3b), and the higher the share of scientists in the research process, the higher the perception of the applicability of research knowledge (H3c). Five hypotheses are evaluated for the variable of applicability according to Bayesian analysis of variance:


$$H_{0}:\mu_{4\,S}=\mu_{3\,S\,1\,E}=\mu_{2\,S\,2\,E}=\mu_{2\,E\,2\,S}=\mu_{3\,E\,1\,S}=\mu_{4\,E}$$

$$H_{3\,a}:\mu_{4\,S}<\mu_{3\,S\,1\,E}<\mu_{2\,S\,2\,E}=\mu_{2\,E\,2\,S}<\mu_{3\,E\,1\,S}<\mu_{4\,E}$$

$$H_{3\,b}:\mu_{4\,S}=\mu_{4\,E}<\mu_{3\,S\,1\,E}=\mu_{2\,S\,2\,E}=\mu_{2\,E\,2\,S}=\mu_{3\,E\,1\,S}$$

$$H_{3\,c}:\mu_{4\,S}>\mu_{3\,S\,1\,E}>\mu_{2\,S\,2\,E}=\mu_{2\,E\,2\,S}>\mu_{3\,E\,1\,S}>\mu_{4\,E}$$

$$H_{u}:\mu_{4\,S},\mu_{3\,S\,1\,E},\mu_{2\,S\,2\,E},\mu_{2\,E\,2\,S},\mu_{3\,E\,1\,S},\mu_{4\,E}$$

The results in Table 5 show the Bayes factor resulting from the ANOVA analysis. As can be seen, *BF*_0*u*_ = 665957.83, that is, the support for *H_0_* is still 665 957.83 times larger than for *H_u_*. It can also be seen that the support for *H*_*3b*_ is 26088.13 times larger than the support for *H*_*u*_. The posterior probabilities are obtained by including *H_u_* in the set of hypotheses examined. They show that *H_0_*, with a posterior probability of 0.96, is the hypothesis with the greatest support and that the preference for*H*_0_ is associated with an error probability of 0.04. Again, as in hypothesis two, we confirm the null hypothesis, i.e., applicability of research knowledge is not dependent on research practice compositions in the research process.

**TABLE 5:** Bayesian informative hypothesis testing (ANOVA) of applicability.

Hypothesis	BF._ɑ_	Posterior probability
*H_0_*	665957.83	0.96
*H*_*3a*_	7.92	0.00
*H*_*3b*_	26088.13	0.04
*H*_*3c*_	7.98	0.00
*H_u_*		0.00

### Summary of Results

Overall, across the varying research team compositions presented, research knowledge is associated with a rather high level of trust, relevance and applicability. Our analyses confirm the assumption that trust in research knowledge is not dependent on research collaboration (Hypothesis 1a). Concerning the question of whether *signals* about a (no) research collaboration *per se* point to practice-relevant and applicable research knowledge, the findings show that practitioners neither perceive signals of practice-relevance nor applicability from any collaborative research setting nor from homogeneous research teams (Hypotheses 2 and 3).

## Discussion

Against the background of the science-practice gap, our study examines whether information about research collaborations between science and practice influences the reception of research findings in the field of adult education. The dimensions examined—trust in the findings as well as assessments of their relevance and applicability—are central prerequisites for the actual use of research findings in the field. Our survey experiment varied compositions of scientists and practitioners in a research team around an identical finding. With reference to considerations based on system and signal theory, our hypotheses suggested that research-practice collaboration and the ratio between participating scientists and practitioners in the research team makes a difference in the reception of the dimensions studied. According to our results, it does not. On average, the descriptive results on trust in the finding, as well as the assessment of its relevance and applicability, show a positive tendency—regardless of the composition of the research team. On the one hand, the results of the Bayesian models support our assumption that trust does not depend on research collaboration. On the other hand, the results of Bayesian models do not support our assumptions about the influence of compositions within research collaborations on reception by signals. Neither the relevance to practice nor the applicability of research findings is influenced by signals about the composition of researchers and practitioners.

### Implications

The results indicate that practitioners have a high level of trust in scientific findings regardless of the composition of the research team. Trust reduces complexity and uncertainty and is therefore a basic prerequisite for scientific knowledge to be translated into decisions and action in the field. An unconditionally high level of trust is generally a good premise for knowledge translation. Are the ivory tower metaphor and sweeping accusations about a lack of practical relevance therefore water under the bridge of the science-practice gap? What are the implications for the discussion on evidence based practice in education?

Trust in science has different degrees of complexity and its conditions are difficult to define (Resnik, 2011). Although our findings indicate that information about research collaboration does not strengthen trust in, nor improve the perception of the relevance and applicability of research knowledge, we cannot conclude that information about research collaboration does not matter because we measured attributions. Research collaboration as a multifaceted element should be considered in further research. After all, research knowledge produced through collaborative research can enrich relevance and applicability as it is integrated with experiential knowledge and situational awareness of complexity in educational practice (van Schaik et al., 2018). To this point, (mostly case) studies (e.g., Coburn and Penuel, 2016; Wentworth et al., 2017) show potential benefits of research collaboration in the specific context of the collaboration (that is those organizations, practitioners and students involved or in close proximity to the research practice partnership).

Our results suggest that cooperation between academics and practitioners in research teams may be beneficial for the realization of research but does not *per se* lead to widespread use of practitioners’ research knowledge. It would be worthwhile to further explore whether and how evidence-based action in small-scale settings can spill over into large-scale knowledge-sharing settings. Against this background, the focus could shift from trust in individual research findings and their origins to trust in media and institutions that act as knowledge brokers to communicate scientific findings to professionals in various educational settings, e.g., clearing houses, practical journals and blogs. Potential spill-over effects of collaborative research results could also be investigated in a more differentiated way with regard to various transfer products from individual projects. Transfer products, such as trainings, textbooks or digital media tailored for practice, are *per se* more application-oriented (Goeze and Schrader, 2011) and could be especially efficient if they were developed in mutual collaboration. The expertise of practitioners on conditions and barriers of the use of research results in the practice of professionals as well as further studies on the user behavior in different professional fields (Henson, 2001; Levin and Rock, 2003) can provide important insights and design information.

### Limitations

Although survey experiments by design promise internal and external validity, by combining experimental designs with representative samples, the application of the method brings limitations and trade-offs for any study based on wbmonitor data. An additional module to the regular question program was enabled, which had a correspondingly limited question program. In form of a classic split-ballot experimental design, the study is limited to the analysis of only one vignette dimension, namely the different composition of actors in a research collaboration. The representation of no or more or less practitioners, respectively, scientists in a research team has only limited informative value for aspects of cooperative research and its potentials. Thus, we are not in a position to test the influence of further relevant characteristics for the description of cooperative research processes (e.g., detailed descriptions of persons involved or descriptions of scope and quality of the involvement) on the reception of scientific knowledge. Likewise, we were unable to vary other factors outside the research collaboration, such as characteristics of the research knowledge (e.g., research methods, scientific language, mode of presentation, etc.).

Furthermore, there is a certain proximity between the two terms “expert” and “scientist” in German. Even though we have added the words “from an adult education institution” to the term “experts,” we cannot rule out the possibility that respondents may also associate the term with a scientific nature. Moreover, future research should take up questions of how the impact of knowledge about collaborations in research processes on reception is influenced by recipients’ characteristics (e.g., general attitudes toward science and/or contextual information on the working environment). Finally, we admit that it cannot be ruled out that the effects could be different in other scientific disciplines. In medical research, for example, cooperation with economically oriented pharmaceutical industries critically affects trust in research. In particular, trust in (educational) science as a complex construct deserves closer examination, which should consider further detailed information of contexts in which research results are produced and contexts in which they are to be used (Mohajerzad and Specht, 2021).

On a more general note, this study counteracts the observation that too little importance is given to the reception of research results (St. Clair, 2004). We think that an expansion of research on the reception of scientific knowledge and its effectiveness in the context of knowledge translation is required. In view of constantly growing empirical research on education, questions of how generated knowledge can be used to inform evidence-based practice in education are receiving relatively little attention. This question should not only be answered conceptually but should also be guided empirically in line with the evidence-informed approach.

## Data Availability Statement

The raw data supporting the conclusions of this article will be made available by the authors, without undue reservation.

## Ethics Statement

Ethical review and approval was not required for the study on human participants in accordance with the local legislation and institutional requirements. The patients/participants provided their written informed consent to participate in this study.

## Author Contributions

AM, JC, and SW contributed to the study conception and design, and performed the material preparation and data collection in cooperation with the Federal Institute for Vocational Education and Training (BIBB). AM and HM performed the data analysis. HM wrote the first draft of the manuscript. AM, JC, SW, and HM wrote sections of the manuscript. All authors commented on the earlier versions of the manuscript, read and approved the final manuscript.

## Conflict of Interest

The authors declare that the research was conducted in the absence of any commercial or financial relationships that could be construed as a potential conflict of interest.

## Publisher’s Note

All claims expressed in this article are solely those of the authors and do not necessarily represent those of their affiliated organizations, or those of the publisher, the editors and the reviewers. Any product that may be evaluated in this article, or claim that may be made by its manufacturer, is not guaranteed or endorsed by the publisher.

## Footnotes

1 Replaced in 2015 by the Every Student Succeeds Act (2015).

2 Tooley and Darby (1998) as cited in Feuer et al. (2002) and Schrader (2014) as quoted in Schrader (2014) also mention such defects.

3 For further information on the survey design: Koscheck and Ohly (2010).

4 Results based on wbmonitor 2018 show following shares for tasks performed by respondents (multiple responses per respondent): planning/management = 87%, teaching/consulting = 29%, administration = 17% (Source. Own calculations using wbmonitor 2018).

5
https://informative-hypotheses.sites.uu.nl/software/bain/

6 The subscript *u* denotes that the means are unrestricted. The Bayesian factors are calculated by integrating so-called posterior and priority distributions with respect to (parts of) Hu (Hoijtink et al., 2019).

7 4S = four Scientists, 3S1E = three Scientists and one Expert, 2S2E = two Scientists and two Experts, 2E2S = two Experts and two Scientists, 3E1S = three Experts and one Scientist and 4E = four Experts.
